# A perspective on *Drosophila* genetics and its insight into human neurodegenerative disease

**DOI:** 10.3389/fmolb.2022.1060796

**Published:** 2022-11-28

**Authors:** Nancy M. Bonini

**Affiliations:** Department of Biology, University of Pennsylvania, Philadelphia, PA, United States

**Keywords:** *Drosophila*, modifier genetics, genetic screens, chaperones, disease models

## Abstract

*Drosophila* has been long appreciated as a classic genetic system for its ability to define gene function *in vivo*. Within the last several decades, the fly has also emerged as a premiere system for modeling and defining mechanisms of human disease by expressing dominant human disease genes and analyzing the effects. Here I discuss key aspects of this latter approach that first intrigued me to focus my laboratory research on this idea. Differences between the loss-of-function vs. the gain-of-function approach are raised—and the insight of these approaches for appreciating mechanisms that contribute to human neurodegenerative disease. The application of modifier genetics, which is a prominent goal of models of human disease, has implications for how specific genes or pathways intersect with the dominant disease-associated mechanisms. Models of human disease will continue to reveal unanticipated insight into fundamental cellular processes—insight that might be harder to glean from classical genetic methodologies vs modifier genetics of disease.

## Introduction

*Drosophila melanogaster*, the simple fruit fly, rose to prominence for its exceptional genetics, for its ability to integrate from genes to chromosomes, and for defining the molecular nature of inheritance. Flies have many morphological landmarks on their external anatomy that are great features for isolating mutations that very specifically and consistently impact the animal—for example, mutations that affect bristle length, bristle number, bristle structure, wing size, wing vein pattern, and so forth. Flies have a rapid lifespan, going from egg to adult fly in about 10 days. Flies are readily crossed for progeny, with unmated individuals being easily isolated, with each mating pair producing hundreds of progeny that can be assessed for genetic markers, mutations and phenotypes. Many techniques of molecular genetics have by now been applied to the fly, allowing one to manipulate nearly any gene in any tissue at any time, for exquisite control of gene function. These features and more have allowed the fly to rise to prominence as a model system for understanding gene function.

In the early 1970s, chromosome wide loss-of-function genetic screens became feasible due to the generation of key *Drosophila* tools like “balancer” chromosomes that allow the isolation and easy maintenance of recessive lethal mutations (Nüsslein-Volhard and Wieschaus, 1980; Wieschaus and Nüsslein-Volhard, 2016). In these screens, the search was for genes whose loss-of-function led to an altered pattern of the larval cuticle. The screens were extraordinary for the insight they ultimately presented: whereas some types of genes could have been predicted, the pattern upon mutation of others was unexpected. The screens launched decades of research on the detailed dance of gene regulation that generates such unanticipated expression patterns, for example stripes in the embryo (Hafen et al., 1984). They also stimulated the idea of similar large scale genetic screens in other model systems to discover the broad set of genes that could be mutated to impact a phenotype of interest, typically for developmental genetics (Mayer et al., 1991; Driever et al., 1996).

These forward genetic screens yielded many landmark discoveries of genes controlling key developmental phenotypes and processes, from the domino of transcription factors that set up and produce the structures of each body segment, to genes involved in orchestrating cellular cytoskeletal morphological changes. This loss-of-function approach, where the normal function of the gene is mutated to be reduced or eliminated, allows understanding of the normal function of that gene. There are additional landmark examples of genes with known functions being discovered through loss-of-function approaches in the fly. For example, the ras signaling pathway in eye development highlighted that the gene, typically found mutated in human cancer/oncogenesis, had a normal function to produce the structure of the eye (Simon et al., 1991; Fortini et al., 1992).

This approach was the one generally used when I started working with *Drosophila* as a postdoctoral scientist and then began my own laboratory.

## Human neurodegenerative disease: A gain-of-function approach

With the sequencing of the fly genome (Rubin et al., 2000), the door opened wide to the idea to focus on a gene based on homology to a human counterpart. Thus one could take a reverse genetic approach. Furthermore, many human diseases that impact the brain with age are dominant disorders associated with gain-of-function mutations and many had been cloned so their gene identify was known (The Huntington’s Disease Collaborative Research Group, 1993; Goate et al., 1991). In polyglutamine diseases, a CAG repeat expansion occurs within the open reading frame of the respective genes, conferring toxicity to the protein and RNA products. Although long CAG repeats were first noted from studies in *Drosophila* (during the cloning of *Notch* (Wharton et al., 1985)), in the fly CAG repeats are not seen to expand on their own and confer a toxic effect as in humans.

There may be many reasons for a lack of spontaneous repeat expansions in the fly: such phenotypes may be dominantly lethal and so would not be isolated; such phenotypes may be variable and selected against by geneticists who favor robust and reproducible phenotypes; such phenotypes could be adult onset and age-dependent as in the case of the human diseases, and the main focus was on development at the time.

The modeling of dominant late-onset human diseases in the fly with expression of the counterpart human disease gene thus introduced a different approach in the fly, whereby those interested in the brain, brain integrity with age, and late-onset human neurological diseases could take advantage of the plethora of molecular genetic approaches of *Drosophila* to understand mechanisms of these human diseases. The phenotypes conferred on the fly by human disease genes can be truly fascinating and marked replicas of the essential features of the human disease, but over an extraordinarily rapid time scale. Moreover, the features of the diseases can include effects that are quite distinct from what had been previously seen upon isolation of classical fly mutations (more below).

Models of the human neurodegenerative diseases are typically generated by upregulation or expression of the disease-associated gene, either normal or with a human familial mutation. Upregulation can be appropriate to model the human disorder because genetic versions of many of these diseases include gene dosage upregulation. For example, increased copy number of *APP* for Alzheimer’s (Wisniewski et al., 1985; Mann and Esiri, 1989) and of alpha-synuclein *SNCA* for Parkinson’s disease (Singleton et al., 2003; Chartier-Harlin et al., 2004; Ibáñez et al., 2004). The polyglutamine diseases, such as Spinocerebellar ataxia type 3 (SCA3) and Huntington’s, are examples of mutation-based diseases due to CAG repeat expansions within the associated genes (Stoyas and La Spada, 2018; Klockgether et al., 2019). These diseases were among the initial diseases of this class of late onset progressive human diseases modeled in the fly (Jackson et al., 1998; Warrick et al., 1998). The genetics of this range of human diseases underscores the validity of modeling these disorders in *Drosophila* by expressing the human gene or human gene with familial mutation.

**FIGURE 1:**
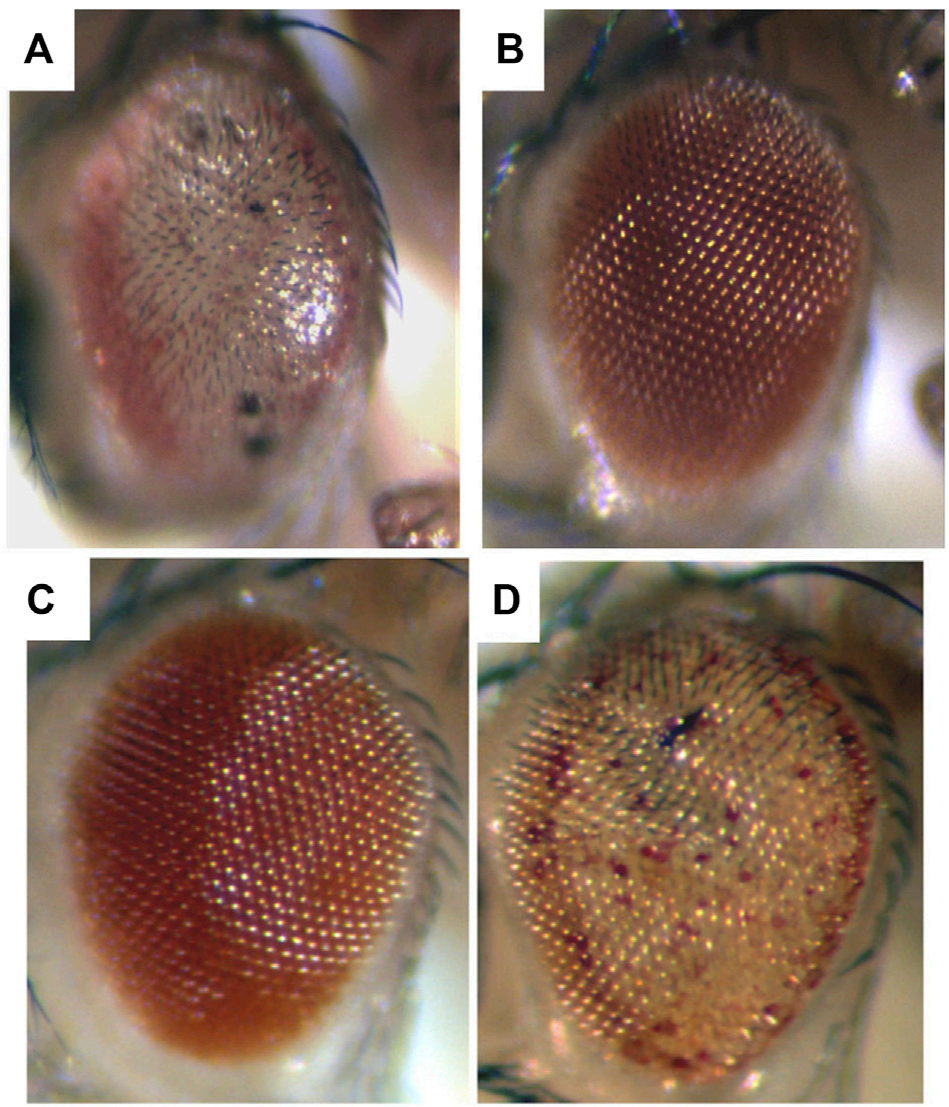
Expression of a toxic human neurodegenerative polyQ protein causes degeneration in the fly, and is modulated by up and downregulation of chaperone activity. Flies expressing **(A)** the toxic Spinocerebellar type 3 (SCA3) disease protein strongly have a very severe degeneration. **(B)** Coexpression with Hsp70 prevents degeneration and the eye returns to normal. By comparison, **(C)** animals expressing the SCA3 disease protein weakly are born with a fairly normal external eye. However **(D)** when a dominant negative form of the Hsc70 chaperone is co-expressed with the disease protein, now the animal has a phenotype similar to severe degeneration (compare to **(A)**). Adapted from (Warrick et al., 1999) with permission.

**FIGURE 2:**
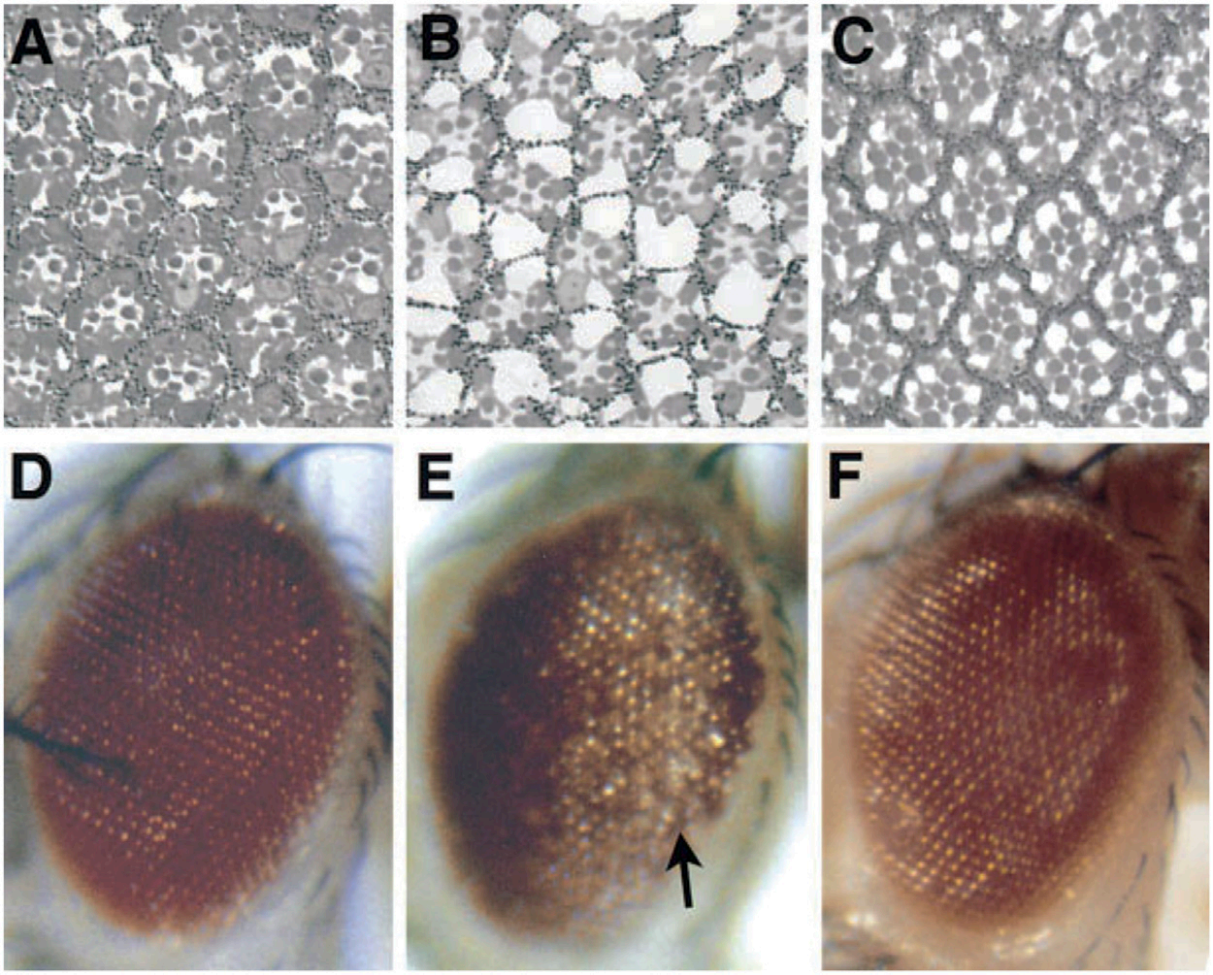
The progressive nature of a disease gene phenotype in *Drosophila*. Expression of a toxic polyglutamine disease protein associated with the human disease SCA3 in the fly eye shows progressive deterioration of the eye with age. When weakly expressed, the protein has minimal effects on the internal **(A)** and external eye **(D)** when the animal first emerges as an adult. **(B,E)** But over just 4 days, the eye undergoes drastic deterioration. **(C,F)** Eye expressing non-toxic protein for comparison, which is normal. From (Warrick et al., 1998) with permission.

To me, among the strikingly distinct and unusual features of expression of the SCA3 polyglutamine human disease protein in the fly was the dramatic degeneration produced (Figure 1A), and the markedly progressive nature of the degeneration. For example, when the disease form of the protein is expressed in the fly eye at a weak level, the animals are born with a rather normal exterior eye; however, the eye undergoes visible loss of external pigmentation and internal retinal deterioration over the next few days of adult life (Warrick et al., 1998) (Figure 2). I found this extraordinary. Alpha-synuclein expression, associated with Parkinson’s phenotypes, leads to dopaminergic neuronal deterioration that occurs progressively with age (Feany and Bender, 2000; Auluck et al., 2002).

**FIGURE 3:**
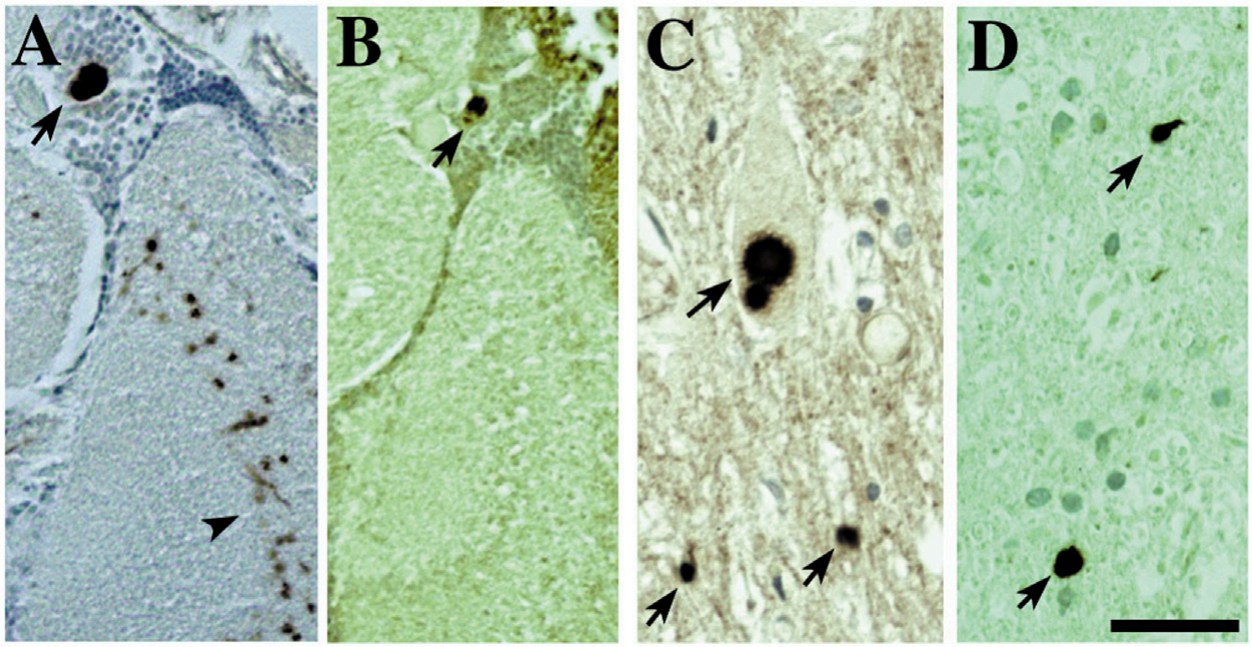
The accumulations of toxic disease proteins in the fly can strikingly resemble the human pathology of the disease. Expression of α-synuclein leads to Lewy-body-like aggregates in the cortex and neurophil that are remarkably similar to the Lewy bodies found in human disease (also (Feany and Bender, 2000; Auluck et al., 2002)). **(A,B)** Fly tissue showing immunostaining of Lewy-body-like aggregates for **(A)** α-synuclein and **(B)** stress-induced Hsp70. Tissue from a Parkinson’s patient showing **(C)** Lewy bodies that immunostain **(D)** for Hsp70. Bar 3 µm. From (Bonini, 2002) with permission.

The feature of progressive degeneration, which is a key characteristic of the human diseases, appears associated with the disease proteins themselves, because simply expression of the genes associated with the disease confers this effect. Disease proteins also form accumulations in fly neurons as they do in human cells that show striking similarities with the features of the human pathology, such as polyglutamine inclusions and alpha-synuclein Lewy bodies (Warrick et al., 1998; Feany and Bender, 2000; Auluck et al., 2002; Bonini, 2002) (Figure 3). This too seems striking, and again indicates that this property of the gene—and of the players in the fly neurons with which the gene interacts to yield the characteristic pathology—is conserved.

In sum, the extent to which the fly recapitulates fundamental features of the counterpart human disease by simple expression of the human disease gene seems remarkable. The fly shows deterioration of tissues (eye, brain, motorneurons), often of the specific cell type (as in alpha-synuclein toxicity to dopaminergic neurons), protein accumulations of the disease protein immunostain with similar factors as in human disease tissue (Figure 3), and similar behavioral effects occur (reduced lifespan, reduced motility, compromised learning and memory). These observations underscore that these features are in essence “captured” and a property of the mutated or upregulated gene itself: simply expressing that gene in the context of a different nervous system (that of the fly) confers these features. This seems a simple conclusion, but is nevertheless a striking point regarding the ability to model human genetic disease in the fly.

## Pathways and processes revealed by modifier genetics

The information learned from modifiers of dominant human disease genes can be in many ways more clear than what can be learned from a detailed study of the loss-of-function of the specific modifier gene on its own. That is, many fundamental cellular pathways like protein folding strikingly modify specific human disease gene phenotypes, yet loss of function of players in these pathways on their own (molecular chaperones, proteasome genes) can be difficult to glean specific insight into as the mutations may be lethal and the phenotype display non-specific, general features. By contrast, as a modifier of the phenotype of a human disease protein, these pathways have proven critically important and unveil their profound importance and functions in this clear context.

Among such players, the first we found—the molecular chaperone Hsp70—is one of the more striking for its ability to mitigate polyglutamine disease. Whereas upregulation of Hsp70 on its own has minimal effects, upregulation of Hsp70 can powerfully mitigate the degeneration associated with a toxic polyglutamine protein and restore a normal fly eye (Warrick et al., 1999) (Figures 1A,B). Importantly, decreasing Hsp70/Hsc70 chaperone activity is also striking in leading to an “acceleration” of the disease effects—that is, it shifts a mild degeneration into a severe degeneration (Figures 1C,D). Dose-sensitive modification by the Hsp70 class of chaperones highlights that the biological impact of the disease gene is very sensitive to the levels of chaperones and protein folding players (proteostasis) in the cell (Warrick et al., 1999; Auluck et al., 2002; Bonini, 2002) and dramatically impacts protein folding ability of the cell (Sinnige et al., 2020; Gidalevitz et al., 2006).

The dose-sensitivity in the fly is underscored by compromised chaperone activity as a risk for human neurodegenerative disease (Wu et al., 2004). Intriguingly, when Elefant and Palter (1999) first examined expression of dominant negative forms of Hsc70 in the fly, the authors noted that the phenotype resembled a degenerative state–but this was difficult to assess until the interaction with human disease proteins was uncovered. Thus, the study of these dominant disease genes in the fly has revealed a whole new depth and impact of these basic biological pathways: whereas chaperone activity was known to be important for protein folding, the striking importance to fundamental disease pathology became appreciated.

These and similar types of findings have dramatically stimulated interest in the regulation of very basic cellular pathways and processes in the brain normally and with age, as age is a prominent risk factor for human neurodegenerative disease. Again, the insight gleaned by interactions with disease proteins has implications well beyond what could have been found by simply studying these genes with the classical loss-of-function approach vs in the context of modifiers of human disease genes.

Modifier genetics though also has limitations or considerations not necessarily evident at the start. For example, having a gene modify a phenotype does not necessarily mean that perturbation of that gene is part of the normal disease process, only that perturbations of that pathway can impact it. One then seeks additional data to determine whether the perturbation is actually a part of the disease process. One also typically examines effects in a tissue of ease: for example, the fly eye. This has advantages because the fly is viable without its eyes, therefore if the protein is toxic leading to death/loss of the cells, one can isolate and study the animal. But the external eye is a pigmented sheath, not neural cells. In addition, if using a reduction of function approach for modifiers, one can only find loss-of-function modifiers for a tissue in which the gene is normally expressed. For example, you might not expect genes that function only at a synapse to necessarily be a modifier of an external eye pigmentation phenotype.

A key point about modifier genetics also is that modifiers can land you as a scientist in entirely new areas of research. A challenge is to then learn about the modifier pathway or process in order to gain insight into how that gene interplays with the disease process. Scientifically this takes one in many directions—examples from my own work include miRNAs (Bilen et al, 2006), epigenetics (Berson et al., 2017), dynamics of transcription (Kramer et al., 2016; Goodman et al., 2019)—each of which is an entire field of research of its own.

One common thread my laboratory ultimately found is that many different modifiers of the disease models we were studying impinge on stress and protein folding pathways. We started our screens searching for modifiers that function in different ways from the chaperones, but were always led back to the stress response. For example, chromatin is re-organized by disease proteins to respond to stress (Berson et al., 2017) and aging impacts the stress response (Liu et al., 2012; Srinivasan et al., 2021).

Given the constant reinforcement of the stress response, the laboratory has recently stepped back and asked a simple question: what actually is the brain acute stress response? Here, surprising we found that the brain fails to display a strong protein chaperone response to acute heat stress, despite a robust transcriptional response (Perlegos et al., 2022). This has led us toward mechanisms contributing to this compromised response, which include epigenetic modification of RNA by m^6^A. This mark is enriched in the fly brain and serves to limit the acute heat stress RNA and protein response.

## The astonishing fly

Perhaps the most striking aspect of the fly as a model is the extent to which what is seen in the fly is also seen in mammals (typically mouse or cell lines), and also inferred to occur in human patient tissue. This is underscored again and again, for chaperone modification of disease phenotypes (Warrick et al., 1999; Auluck et al., 2002), chromatin modification of disease phenotypes (Frost et al., 2014; Berson et al., 2017; Nativio et al., 2020), specific protein interactions (Elden et al., 2010; Freibaum et al., 2015), and others. Thus, the fly can be a less expensive and genetically tractable way to explore interactions of relevance to disease. One limitation is that there can be a trade-off lack of depth into any one pathway because efforts must be expended into demonstrating the conservation to other systems.

By contrast to the above, for many processes in the fly, like mechanisms of neural development (Konstantinides et al., 2022) or study of the clock (Toda et al., 2019), tangential data that the precise pathway or process revealed also occurs in mammals and humans is not required. Accepting that what the fly reveals will be reflected in other systems, including human and human disease, and then focusing in depth on what the fly can reveal might be considered an advance in the field—depth vs breadth to establish that there are signs of a similar process going awry in mouse and human models. Regardless, an impressive body of work has been generated showing how the fly has uncovered new pathways and mechanisms of great relevance to human disease (see Shulman et al., 2003; Marsh and Thompson, 2006; Casci and Pandey, 2015; McGurk et al., 2015; Verheyen, 2022 for reviews).

Another impressive feature of the fly is the way in which the animal can so accurately reflect different disease situations, including some that *Drosophila* normally does not encounter, to allow extraordinary genetic insight. Models beyond neurodegenerative disease include cancer and cancer metastasis (Pagliarini and Xu, 2003; Enomoto et al., 2018; Liu et al., 2022), autism (Tian et al., 2017; Dahlhaus, 2018), regeneration (Coupe and Bossing, 2022; Nagai et al., 2022), kidney disease (Dow et al., 2022), metabolic disease (Liguori et al., 2021; Moraes and Montagne, 2021), and sleep (Chakravarti et al., 2017).

Given this is a perspective, in these studies I have learned that genetics is fascinating—both fly and human (and it was an excellent type of research I could do while also being a professor, as I could be interrupted). It becomes important to engage in screens all the time–new types of screens and with new approaches. Screens of different types also means one can select to pursue the mechanisms and genes that make sense, and circle back later to others that prove enigmatic at the time. Screens include alternative approaches like RNA-seq and other molecular approaches, for a different way of looking at the genome in the disease and modified situation. Altogether, modifier genetics presents intriguing puzzles that can be quite distinct from standard genetics.

Current and future application of additional approaches and technologies will reveal even more secrets of the complexity of the disease situation. For example, the glial response has emerged as highly impactful to human degenerative disease. The fly has a range of glial types, and one can sort neurons from glia or perform single cell analyses or ribo-seq to define specific events in individual or subset of cells (Chen and Dickman, 2021, 2017; Yildirim et al., 2019; Sheng et al., 2020). Thus the future holds great promise for uncoupling key events and processes impacted upon disease that can be studied and teased out in great detail in the fly for the potential benefit of therapeutics. The collective approaches and body of work from the *Drosophila* community as a whole underscores the influence and capability of the fly, and the energetics, enthusiasm and creativity of the scientists who choose this field of research.

## Data Availability

The original contributions presented in the study are included in the article/supplementary material, further inquiries can be directed to the corresponding author.
